# Risk of psychiatric disorders following trigeminal neuralgia: a nationwide population-based retrospective cohort study

**DOI:** 10.1186/s10194-015-0548-y

**Published:** 2015-07-15

**Authors:** Tung-Han Wu, Li-Yu Hu, Ti Lu, Pan-Ming Chen, Hon-Jhe Chen, Cheng-Che Shen, Chun-Hsien Wen

**Affiliations:** Department of Psychiatry, Kaohsiung Veterans General Hospital, Kaohsiung, Taiwan; Division of Chest Medicine, Department of Internal Medicine, Taichung Veterans General Hospital, Chiayi Branch, Chiayi, Taiwan; School of Medicine, National Yang-Ming University, Taipei, Taiwan; Department of Psychiatry, Yuanshan & Su’ao Branch, Taipei Veterans General Hospital, Yilan, Taiwan; Department of Family Medicine, Kaohsiung Veterans General Hospital, Kaohsiung, Taiwan; Department of Information Management, National Chung-Cheng University, Chiayi, Taiwan; Department of Psychiatry, Chiayi Branch, Taichung Veterans General Hospital, Chiayi, Taiwan; Department of Anesthesiology, Kaohsiung Veterans General Hospital, No. 386, Ta-Chung 1st Rd., Tzuo-Yin Dist., Kaohsiung City, 81362 Taiwan

**Keywords:** Trigeminal neuralgia, Depressive disorder, Anxiety disorder, Sleep disorder, Retrospective cohort study

## Abstract

**Background:**

TN is one of the most common causes of facial pain. A higher prevalence of psychiatric co-morbidities, especially depressive disorder, has been proven in patients with TN; however, a clear temporal-causal relationship between TN and specific psychiatric disorders has not been well established. We performed a nationwide population-based retrospective cohort study to explore the relationship between TN and the subsequent development of psychiatric disorders, including schizophrenia, bipolar disorder, depressive disorder, anxiety disorder, and sleep disorder.

**Methods:**

We identified subjects who were newly diagnosed with TN between January 1, 2000 and December 31, 2010 in the Taiwan National Health Insurance Research Database. A comparison cohort was constructed for patients without TN who were matched according to age and sex. All TN and control patients were observed until diagnosed with psychiatric disorders, death, withdrawal from the National Health Institute system, or until December 31, 2010.

**Results:**

The TN cohort consisted of 3273 patients, and the comparison cohort consisted of 13,092 matched control patients without TN. The adjusted hazard ratio (aHR) of depressive disorder, anxiety disorder and sleep disorder in subjects with TN was higher than that of the controls during the follow-up [aHR: 2.85 (95 % confidence interval: 2.11–3.85), aHR: 2.98 (95 % confidence interval: 2.12–4.18) and aHR: 2.17 (95 % confidence interval: 1.48–3.19), respectively].

**Conclusions:**

TN might increase the risk of subsequent newly diagnosed depressive disorder, anxiety disorder, and sleep disorder, but not schizophrenia or bipolar disorder. Additional prospective studies are required to confirm these findings.

## Background

Trigeminal neuralgia (TN) is a common cause of chronic orofacial pain. However, TN may also be one of the most puzzling orofacial pain conditions and affected patients are often difficult to treat [1, 2]. Typically, TN is characterized by recurrent brief episodes of unilateral electric shock-like pain, followed by asymptomatic periods without pain, in the distribution of one or more divisions of the trigeminal nerve that are triggered by innocuous stimuli [3]. However, in some patients a constant dull background pain may persist. Therefore, based on the clinical features, TN is categorized into typical and atypical form, the latter characterized by a constant background pain and, sometimes, sensory disturbances in the affected division [1].

The annual incidence of TN is 4 to 13 per 100,000 people [4, 5]. Women are more often affected than men, with an approximate male to female ratio of 1:1.7 [6]. This female predominance may be related to the increased longevity of women compared to men. To date, the precise mechanism for TN remains unclear. Among a variety of causes of TN [7–9], the microvascular compression hypothesis is the most popular, but the origin and pathogenesis of TN remains controversial [2, 10].

Chronic diseases and pain, such as migraine, are frequently accompanied by psychiatric abnormalities [11]. Numerous studies have tried to clarify the associations between the migraine and psychiatric disorders in the past decade [12–14]. However, recent years have seen increased attention being given to the relationship between TN and psychiatric disorders such as depression, anxiety, or sleep disturbance [15–17]. In addition, in the past decade, there has been renewed interest in chronic inflammation, which is also associated with the pathophysiology of TN development [18–20]. Studies have shown that cytokines circulating in the plasma may impair the function of the blood–brain barrier (BBB) [21], which may indicate that peripheral inflammation is associated with the upregulation of CNS inflammation [22]. The missing link between peripheral and CNS inflammation, that is, the disruption of BBB, may play a vital role in the development of schizophrenia [23] and bipolar disorder [24]. Moreover, it is crucial to remember that chronic TN may result in psychiatric morbidities and reduced quality of life, in addition, existence of newly-diagnosed psychiatric disorders could be seen as predictors of poorer treatment outcome among the patients with TN [25].

In 2014, Meskal et al. reported that patients with TN may be at risk for cognitive impairments [26], which resemble those of patients with chronic pain conditions [27–29]. However, apart from TN, other variables such as the medicines used to treat TN (e.g., anticonvulsants), pain-related depressive or anxiety symptoms, and the other factors that decrease cognitive reserve such as advanced age, multiple sclerosis or other degenerative disease of the nervous system may be alternative explanations for cognitive deficits among patients with TN [28, 30, 31]. Nevertheless, whether TN itself is an independent risk factor for subsequent cognitive impairments or not, the cognitive impairments have been shown to affect therapy adherence, personal relationships, daily functioning, capacity for work and leisure activities, and quality of life in patients with TN [32, 33]. In 2014, He et al. demonstrated that patients with TN exhibit increased attention toward pain-related information and this phenomenon may be associated with the development of anxiety and depression [34]. For sleep investigations, although many clinicians who treat TN report that patients are rarely awakened at night by pain attacks, Devor et al. discovered that about 60 % of the patients with TN had painful awakenings at least occasionally and emphasized that painful awakenings among patients with TN are in fact quite common [17].

Although the above-mentioned research has provided insight into the association between TN and co-morbid psychiatric disorders, most of these study results were based on a cross-sectional study design and lacked a longitudinal perspective. Additionally, psychiatric disorders in these studies were often evaluated using rating scales, such as Beck Depression Inventory, Self-Rating Depression Scale, Pain Anxiety Symptoms Scale and Self-Rating Anxiety Scale, rather than a clinical diagnosis by a psychiatrist. Furthermore, the small sample size of these studies provided low statistical power.

To date, national data and large-scale studies regarding the association between TN and risk of subsequent psychiatric disorders are lacking. Due to this fact and based on the hypothesis that patients with TN may have a higher risk of developing psychiatric disorders, especially depressive, anxiety, and sleep disorder, we designed a nationwide population-based retrospective cohort study to explore the possible link between these TN and specific psychiatric disorders.

## Methods

### Data sources

Our data set was derived from the Taiwan National Health Insurance Research Database (NHIRD) (http://nhird.nhri.org.tw/en/index.html). In 1995, the National Health Insurance (NHI) program, a government-run insurance program with a single-payer insurance system, was established in Taiwan. By December 2010, more than 23 million people were enrolled nationwide, with a coverage rate of 99.6 % [35]. This database includes the entire patient registry and claims data from this health insurance system, ranging from demographic data to detailed orders from ambulatory and inpatient care. All data were de-identified by encrypting the identification codes of patients and medical facilities so that no individual could be identified during the investigation or processing of the database. Personal information, such as body weight, height, laboratory findings, lifestyle, and smoking and alcohol habits, was not available in the NHIRD [36]. Data used to perform the analyses conducted in this study were retrieved from the Longitudinal Health Insurance Database 2000 (LHID 2000), a subset of the NHIRD. LHID 2000 is a data set released by the NHRI that contains all original claims data for 1 million randomly selected beneficiaries in the 2000 Registry of Beneficiaries.

### Ethics statement

The Institutional Review Board of Kaohsiung Veterans General Hospital approved this study. Written consent from the patients was not obtained, because the NHI dataset consists of de-identified secondary data for research purposes and the Institutional Review Board of Kaohsiung Veterans General Hospital issued a formal written waiver of the need for consent.

### Study population

Using data extracted from the LHID 2000, we conducted a retrospective cohort study of patients who were newly diagnosed with TN (ICD-9-CM code 350.1) between January 1, 2000 and December 31, 2010. In order to ensure diagnostic validity and patient homogeneity, only patients who were diagnosed with TN by a neurologist and had at least two consensual TN diagnoses were selected for the study group. The index date was defined as the date of patients with TN who were enrolled in our study according to the selection criteria mentioned above. We excluded patients who were diagnosed with TN between January 1, 1996, and December 31, 1999. We also excluded patients who were diagnosed with psychiatric disorders (A codes: A210-A219; ICD-9-CM codes: 290–319) before they were diagnosed with TN. For each patient with TN included in the final cohort, four age- and sex-matched comparison patients with the same index date and without a history of psychiatric illness were randomly selected from the LHID 2000. The primary clinical outcomes assessed were schizophrenia, depressive disorder, bipolar disorder, anxiety disorder, and sleep disorder. Moreover, in order to enhance the reliability of the diagnosis of psychiatric disorders, patients of diagnosed psychiatric disorders based on the ICD-9-CM code in the database were included only when the diagnostic process or assessment was performed by a qualified psychiatrist, which information has also been record in the same database. Therefore, all TN and comparison patients were observed until diagnosed with schizophrenia (ICD-9-CM code: 295), depressive disorder (ICD-9-CM codes: 296.2, 296.3, 300.4, and 311), bipolar disorder (ICD-9-CM codes: 296.0, 296.1, 296.4, 296.5, 296.6, 296.7, 296.8, 296.80, and 296.89), anxiety disorder (ICD-9-CM codes: 300.0, 300.2, 300.3, 308.3, and 309.81), or sleep disorder (ICD-9-CM codes: 780.5, 307.4 [excluding 780.51, 780.53, 780.57]) by a psychiatrist, or until death, withdrawal from the NHI system, or until December 31, 2010.

### Statistical analysis

The incidence of newly diagnosed schizophrenia, depressive disorder, bipolar disorder, anxiety disorder, or sleep disorder in the TN and comparison patients was calculated, and independent *t* tests and chi-squared tests were conducted to examine the differences in the characteristics between the TN and comparison patients. A Cox proportional-hazards regression model was constructed to calculate the hazard ratio (HR) of schizophrenia, depressive disorder, bipolar disorder, anxiety disorder, and sleep disorder of the TN cohort and comparison cohort. In addition, we performed a multivariate Cox proportional-hazards regression model to adjust for possible confounding variables that might have an effect on the risk of depressive, anxiety, and sleep disorders. Control variables, such as age; sex; common co-morbidities including hypertension, diabetes mellitus, dyslipidemia, coronary artery disease, congestive heart failure, chronic pulmonary disease, and malignancy; urbanization; and monthly income were included as covariates in the univariate model. Factors that demonstrated a moderately significant statistical relationship in the univariate analysis (*P* < .1) were selected in a multivariate Cox proportional-hazards regression model. Subgroups were stratified according to the duration since TN diagnosis after calculating the incidence rates (per 1000 person-year) of depressive, anxiety, and sleep disorders, and the risk ratios in order to investigate potential surveillance bias.

We used SAS statistical software for Windows, Version 9.3 (SAS Institute, Cary, NC, USA), for data extraction, computation, linkage, processing, and sampling. All other statistical analyses were performed using the SPSS statistical software for Windows, Version 20 (IBM, Armonk, NY, USA). The results of comparisons with a *P* value less than .05 were considered to indicate a statistically significant relationship.

## Results

Our study sample comprised 3273 patients with TN (61.7 % male) and 13,092 control patients without TN. The comparisons of the demographic and clinical variables between the TN and control patients are presented in Table 1. The median age of the patients with TN group was 45.6 years (interquartile range, 33.9 to 54.8 y) and the median follow-up duration was 3.08 years (interquartile range, 1.92 to 3.98 y). A higher percentage of patients with TN were observed in the group of people aged 40–59 years.

**Table 1:** Characteristics of TN and psychiatric illness and comparison subjects

		TN and psychiatric illness	Control cohort	*P* values
No.		3273	13,092	
Age (years)^a^		45.6 (33.9–54.8)	45.6 (33.9–54.8)	0.999
Distribution of age				
	20–39	1299 (39.7)	5196 (39.7)	0.999
	40–59	1423 (43.5)	5692 (43.5)	0.999
	≥60	551 (16.8)	2204 (16.8)	0.999
Sex				
	Female	1255 (38.3)	5020 (38.3)	0.999
	Male	2018 (61.7)	8072 (61.7)	
Comorbidities				
	Hypertension	725 (22.2)	2192 (16.7)	<0.001^*^
	Diabetes mellitus	442 (13.5)	1285 (9.8)	<0.001^*^
	Dyslipidemia	717 (21.9)	1795 (13.7)	<0.001^*^
	Coronary artery disease	25 (0.8)	91 (0.7)	0.650
	Congestive heart failure	71 (2.2)	226 (1.7)	0.090
	Cerebrovascular disease	134 (4.1)	421 (3.2)	0.014
	Chronic pulmonary disease	591 (18.1)	1318 (10.1)	<0.001^*^
	Malignancy	48 (1.5)	213 (1.6)	0.581
Income				<0.001^*^
	Low income	1199 (36.6)	5732 (43.8)	
	Medium income	1315 (40.2)	4982 (38.1)	
	High income	759 (23.2)	2378 (18.2)	
Degree of urbanization				0.072
	Urban	2112 (64.5)	8172 (62.4)	
	Suburban	938 (28.7)	3940 (30.1)	
	Rural	223 (6.8)	980 (7.5)	
Follow-up, years^a^		3.08 (1.92–3.98)	3.08 (1.92–3.99)	0.852
Newly diagnosed psychiatric disorders, N (%)				
	Schizophrenia	3 (0.09)	5 (0.04)	1.000
	Bipolar disorder	1 (0.09)	6 (0.05)	0.203
	Depressive disorder	73 (2.23)	103 (0.79)	<0.001^*^
	Anxiety disorder	59 (1.80)	79 (0.60)	<0.001^*^
	Sleep disorder	40 (1.22)	74 (0.57)	<0.001^*^

^a^Median (interquartile range)

^*^Statistical significance

During the follow-up period, 176 (5.4 %) patients with TN and 267 (2.0 %) comparison patients were diagnosed with psychiatric illnesses. The most common subsequent psychiatric illnesses among the patients with TN were depressive disorder in 73 patients (2.23 %), anxiety disorder in 59 patients (1.80 %), and sleep disorder in 40 patients (1.22 %). Overall, significantly higher incidences of depressive disorder (*P* < .001), anxiety disorder (*P* < .001), and sleep disorder (P < .001) were observed in the patients with TN than in the comparison patients.

After adjusting for age, sex, co-morbidities, urbanization, and monthly income, the results indicated that patients with TN exhibited a markedly higher risk for subsequent depressive disorder (adjusted hazard ratio [aHR] 2.85, 95 % confidence interval [CI] 2.11–3.85), anxiety disorder (aHR 2.98, 95 % CI 2.12–4.18), and sleep disorder (aHR 2.17, 95 % CI 1.48–3.19) (Table 2).

**Table 2:** Hazard ratios of time until psychiatric illness between TN and comparison subjects during a 10-year follow-up period

	Crude HR (95 % CI)	Adjusted HR (95 % CI)^b^
Schizophrenia	2.40 (0.35–10.03)	2.40 (0.35–10.03)
Bipolar disorder	0.67 (0.08–5.55)	0.69 (0.08–5.76)
Depressive disorder	2.87 (2.13–3.87)^a^	2.85 (2.11–3.85)^a^
Anxiety disorder	3.02 (2.15–4.23)^a^	2.98 (2.12–4.18)^a^
Sleep disorder	2.18 (1.48–3.20)^a^	2.17 (1.48–3.19)^a^

*HR* hazard ratio; *CI* confidence interval

^a^Statistical significance

^b^Adjusted for age, sex, hypertension, diabetes mellitus, dyslipidemia, coronary artery disease, congestive heart failure, cerebrovascular disease, chronic pulmonary disease, malignancy, income and urbanization

A sub-analysis based on the duration of follow-up revealed that the risk of newly diagnosed depressive, anxiety and sleep disorders were significantly elevated not only within the first year but also more than 1 year following a TN diagnosis. The results of this sub-analysis are summarized in Table 3.

**Table 3:** Number of newly diagnosed depressive, anxiety and sleep disorders between TN and comparison subjects which was stratified by follow-up duration

Follow-up duration (year)	TN and psychiatric illness		Control cohort		Risk ratio (95 % CI)
	No. of Depressive disorder	Per 1000 person-years	No. of Depressive disorder	Per 1000 person-years	
Overall	73	2.76	103	0.82	2.86 (2.09–3.90)^a^
0–1	32	9.85	24	1.84	5.36 (3.06–9.51)^a^
≥1	41	3.93	79	1.94	2.02 (1.33–3.02)^a^
Follow-up duration (year)	TN and psychiatric illness		Control cohort		Risk ratio (95 % CI)
	No. of Anxiety disorder	Per 1000 person-years	No. of Anxiety disorder	Per 1000 person-years	
Overall	59	2.57	79	0.85	3.01 (2.11–4.27)^a^
0–1	20	6.14	20	1.53	4.01 (2.05–7.86)^a^
≥1	39	4.02	59	1.46	2.76 (1.79–4.22)^a^
Follow-up duration (year)	TN and psychiatric illness		Control cohort		Risk ratio (95 % CI)
	No. of sleep disorder	Per 1000 person-years	No. of sleep disorder	Per 1000 person-years	
Overall	40	1.74	74	0.80	2.17 (1.44–3.24)^a^
0–1	12	3.68	20	1.53	2.40 (1.07–5.16)^a^
≥1	28	2.88	54	1.38	2.09 (1.27–3.35)^a^

CI confidence interval; ^a^Statistical significance

## Discussion

In our study, a nationwide retrospective cohort study was performed to investigate the hazard ratio of newly diagnosed psychiatric disorders following a diagnosis of TN between the patients with TN and the comparison cohort without TN. The main finding of our study yielded an aHR of depressive disorder that was 2.85 times greater for patients with TN than for the comparison cohort (Table 2). We also found that subjects with TN were more likely to develop anxiety and sleep disorders than the controls during the follow-up period. Furthermore, our analysis showed that hypertension, diabetes mellitus, dyslipidemia and chronic pulmonary disease were more prevalent in patients with TN than in patients without TN (Table 1), which is consistent with the results of previous studies, with the exception of chronic pulmonary disease [37, 6, 38–41].

Based on our results, the patients with TN exhibited no higher risk for subsequent schizophrenia and bipolar disorder. According to the update guidelines, carbamazepine (CBZ) is the first-line medical treatments for pain control in patients with TN [42]. The initial number of responders was 98 % with CBZ at a median dosage of 600 mg (range 200–1200 mg) [43]. However, based on the perspective of psychiatry, since the early 1970s, CBZ has also been used in the treatment of bipolar disorder, both in acute mania and maintenance therapy. In addition, CBZ was found to have strong preclinical data for the treatment of co-morbid bipolar disorder and chronic pain [44]. Therefore, the association between TN and bipolar disorder may be underestimated under the CBZ use.

Several studies have confirmed that depression and anxiety are the most common co-morbidities in patients with TN [45, 34, 46]. Our results indicate that TN might be a risk factor for subsequent depressive and anxiety disorders. There are several possible explanations for these results regarding increased risk of new-onset depressive and anxiety disorders in patients with TN. First, TN may cause a dysregulation of central monoaminergic neurotransmitters, such as a decrease in serotonin and norepinephrine, which is related to the development of depressive and anxiety disorders [47, 48]. In 1997, Strittmatter et al. observed that the concentrations of both norepinephrine and serotonin metabolites were significantly decreased in the CSF of patients with TN. In addition, the decrease of these monoaminergic neurotransmitters in the CSF showed a significant negative correlation with the duration of TN [49]. Second, a link has been demonstrated between TN and chronic inflammation. Most of patients with TN also suffered from chronic maxillary sinusitis, periodontitis, periostitis, phlegmon, and dental cysts [18], and some studies indicate that these chronic inflammation disorders may be a direct cause of TN development [19, 20]. Recent studies have also shown that chronic inflammation plays a critical role in the pathophysiology of schizophrenia, bipolar disorder, depressive disorder, and anxiety disorders [50, 23, 24, 51]. Peripheral chronic inflammation disorders that cause TN may take longer duration to induce similar chronic inflammation in the central nervous system [18]. Third, Devor et al. proposed the ignition hypothesis in 2002 as a possible etiology of trigeminal neuralgia [52, 53]. According to this hypothesis, TN results from specific abnormalities of trigeminal afferent neurons in the trigeminal root or ganglion. Injury renders axons and their somata hyperexcitable. These hyperexcitable afferents, in turn, give rise to pain paroxysms as a result of synchronization after discharge activity [52]. Studies have proved that glutamate might play an important role during these synchronizations through *N*-methyl-D-aspartate (NMDA) receptors [54, 55]. In addition, glutamate is a key neurotransmitter involved in both the peripheral and central nervous systems. For example, glutamate produces central sensitization of trigeminal as well as spinal cord dorsal horn neurons [56, 57]. From the psychiatry perspective, glutamate is the most widely used excitatory transmitter in the central nervous system, and several studies in animals and humans have suggested that it may increase the risk of developing depressive and anxiety disorders [58, 59, 50]. Therefore, glutamate antagonists or drugs that target NMDA receptors may be beneficial in treating depressive and anxiety disorders [60–62]. In short, the role of glutamatergic systems in the pathophysiology of TN may increase the risk of subsequent depressive and anxiety disorders.

Patients with TN often report sleep disturbances, with awakenings caused by pain attacks. In a study of sleep disturbance assessed by the questionnaires originated at a conference of the Australian Trigeminal Neuralgia Association, over half of the patients with TN experienced painful awakenings often or occasionally [17]. In our study, patients with TN were found to have a 2.17 times greater risk of developing a sleep disorder than the comparison cohort. However, only 1.22 % of patients with TN have a sleep disorder. The wide range in the prevalence of sleep disorders in studies of TN is likely due to the different methods used for measuring sleep disorders.

In the present study, we conducted a subgroup analysis stratified according to the duration between the diagnosis of TN and new-onset psychiatric illnesses (Table 3). The results indicated that incidence of newly-diagnosed depressive, anxiety and sleep disorders were increased not only within the first year but also more than 1 year after a diagnosis of TN. Patients with TN are likely to exhibit a higher frequency of outpatient visits than the general population, leading to an earlier diagnosis of psychiatric illnesses which may cause surveillance bias. To exclude this possibility, we conducted a subgroup analysis stratified according to the duration between the diagnosis of TN and new-onset psychiatric disorders. The results indicated that incidence of depressive, anxiety, and sleep disorders still increased after the first year following a diagnosis of TN. Hence, concluding that the increased risk of depressive disorder, anxiety disorder, and sleep disorder among patients with TN in the current study was not due to surveillance bias.

To our knowledge, this is the first large population-based study to investigate the association between TN and subsequent psychiatric disorders in Asian area. Large sample size, a maximum follow-up period of 10 years, and improving reliability of diagnosis by specialists constitute the strengths of our study. Additionally, our study design included an unbiased participant selection process. In addition, participation in the Taiwan NHI is mandatory and all residents of Taiwan can access health care with low copayments, therefore, referral biases are low and follow-up compliance is high.

Certain limitations inherent to the use of health insurance claims databases must be considered. First, the results of laboratory surveys and patient’s symptoms could not be obtained from the database. Consequently, the influence of different types of TN and its severity as risk factors for developing subsequent psychiatric disorders could not be determined. Second, the NHIRD does not provide detailed information on patients such as tobacco use, alcohol consumption, body mass index, major life events, and family history of psychiatric illnesses. These conditions are all major risk factors for the development of psychiatric illnesses. Therefore, we were unable to control for these potentially confounding factors. Third, the diagnosis of TN was identified using the ICD-9 codes from the database, and its prevalence may be underestimated because only subjects seeking medical evaluation can be identified; however, this would most likely result in an underestimate of the association between TN and psychiatric illnesses. Finally, the diagnoses in NHI claims are primarily for administrative billing, and do not undergo verification for scientific purposes. Therefore, validation study of NHIRD for patients with some frequently seen diagnoses may play an essential role on improving the quality of database study in the future.

## Conclusions

Our nationwide population-based retrospective cohort study provides further evidence of an excessive risk of depressive, anxiety, and sleep disorders among patients with TN. Depressive, anxiety, and sleep disorders are treatable psychiatric illnesses and have great impact on the quality of life for patients with TN. Therefore, clinicians should be alerted to the possibility of patients with TN developing depressive, anxiety, or sleep disorders.

## Abbreviations

TN: Trigeminal neuralgia
aHR: adjusted hazard ratio
NHIRD: National Health Insurance Research Database
NHI: National Health Insurance
LHID: 2005 Longitudinal Health Insurance Database 2005
HR: Hazard ratio
CSF: Cerebrospinal fluid
NMDA: N-methyl-D-aspartate
